# Changes in Patellar Morphology Following Soft Tissue Surgical Correction of Recurrent Patellar Dislocation in Children with Low‐Grade Trochlear Dysplasia

**DOI:** 10.1111/os.13193

**Published:** 2022-07-11

**Authors:** Cong‐lei Dong, Yan‐yang Wang, Wei Lin, Xiao‐bo Chen, Chen‐yue Xu, Mao‐zheng Wei, Fei Wang

**Affiliations:** ^1^ Department of Orthopaedic Surgery Third Hospital of Hebei Medical University Shijiazhuang China; ^2^ Department of Imaging Third Hospital of Hebei Medical University Shijiazhuang China

**Keywords:** Children, CT scan, Patellar morphology change, Recurrent patellar dislocation, Trochlear dysplasia

## Abstract

**Objective:**

To investigate the changes in patellar morphology following soft tissue surgical correction of recurrent patellar dislocation in children with low‐grade trochlear dysplasia.

**Methods:**

The prospective cohort study was performed between November 2007 and December 2012. Finally, 25 cases, with the mean age of 8.4 years (range from 7 to 10 years), were admitted to the study. All patients were diagnosed as bilateral recurrent patellar dislocation associated with femoral trochlear dysplasia. The knee that suffered injury or was dislocated was treated with medial patellar retinacular plasty (surgery group). The contralateral knee, which served as a control, was treated conservatively (conservative group). Axial CT scans were undertaken in all patients to assess the patellar morphological characteristics.

**Results:**

The mean follow‐up time was 60.8 months (range 48 to 75 months). Preoperatively, there were no statistically significant differences between the patellar morphology in the two groups (*P* > 0.05). Many radiological parameters of patellar morphology were significantly different between the two groups at the final follow‐up, including well‐known parameters, such as the mean patellar width (surgery group, 40.58 mm [SD 1.26]; conservative group, 36.41 mm [SD 1.17]; *P* < 0.05), the mean patellar thickness (surgery group, 11.59 mm [SD 0.74]; conservative group, 9.38 mm [SD 0.56]; *P* < 0.05) and the mean Wiberg index (surgery group, 0.54 [SD 0.06]; conservative group, 0.72 [SD 0.08]; *P* < 0.05). There are also little‐known parameters, such as the ratio of length of lateral patella to medial patella (surgery group, 1.26 [SD 0.17]; conservative group, 1.69 [SD 0.21]; *P* < 0.05), which was a measurement of facet asymmetry. However, the Wiberg angle was not significantly different between the two groups (surgery group, 128.63° [SD 9.05]; conservative group, 125.47° [SD 13.96°]; *P* > 0.05) at the final follow‐up. No complications were found.

**Conclusions:**

The patellar morphology can be significantly improved by early soft tissue surgical correction in children with patellar instability associated with low‐grade femoral trochlear dysplasia.

## Graphical abstract

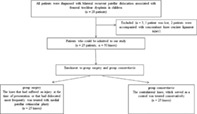

## Introduction

The patella is the largest sesamoid bone in the body, and the femoral trochlear keeps the patella in orbit for normal knee movement^1^. The patellofemoral joint, as the name suggests, is the joint of the patella and the femur. Among them, the patella can increase the distance between the knee flexion and extension axis and the knee extension device, which is conducive to knee extension activity, so the normal patellofemoral joint structure is very important^2^. Patellar dislocation is a disease of pathological dislocation of patella from patellofemoral joint. The age group affected is mainly 10–16‐year‐olds, mostly female, with an incidence of about 5.8/100,000. The etiology involved a variety of factors, such as acute trauma, chronic ligament relaxation, bone misalignment, connective tissue disease or anatomical pathology. Over time, patients with patella dislocation developed debilitating pain, basic functional limitations, and arthritis^3^. If the child suffered from patella dislocation, it will not only bring great pain to the body, but also affect their normal life and ability to walk in the future^4^. In view of the great harm of patella dislocation, there are many studies on patella dislocation, which include not only clinical but also basic experimental studies. Some studies have shown that its occurrence may be related to the dysplasia of femoral trochlea^5^. Correspondingly, patellar dislocation can also alter the shape of the femoral trochlea, especially in developing youth, and the association with trochlea dysplasia was reciprocal^6^. It is known that mechanical stress can affect bone formation and development^7^, ^8^. There is also clinical evidence that, for example, dislocation of the hip joint may lead to acetabular dysplasia^9^. It has been reported that children with both acetabular dysplasia and dislocation of the hip can have their joint shape returned to normal after reduction, possibly through remodeling^10^, ^11^. The shoulder joint is similar^12^, ^13^. This suggested that correction of articular alignment early (before epiphyseal closure) can affect the development and structure of the corresponding bone.

Similar to the hip and shoulder joints mentioned above, in the patellofemoral joint, the stress passed from the articular cartilage to the subchondral bone, and then to the cancellous and cortical bone. This transfer of load can stimulate the growth and remodeling of the patella and femur^8^. The unique concave‐convex matching between patella and femoral trochlea was the structural basis of its biomechanical function^14^. Previous studies showed that early patellar instability can lead to trochlear dysplasia, and early patellar displacement can prevent the development of trochlear dysplasia in growing rabbits^6^, ^15^. In addition, in children with recurrent patellar dislocation complicated by femoral trochlear dysplasia, early soft‐tissue surgery (before epiphyseal closure) may improve femoral trochlear morphology^16^, ^17^. There are many surgical treatments for patellar dislocation, and the most common and effective one was reconstruction of the medial patellofemoral ligament plus release of the lateral patellofemoral ligament. The medial patellofemoral ligament was the most important ligament to prevent lateral dislocation of the patella, providing 53%–60% of the medial tension. The decrease of tension after medial retinacular injury was the most important factor for recurrence of patella dislocation^18^.

So the patella, which also makes up the patellofemoral joint, is also affected by this anomaly of alignment. The abnormality of patella shape has strong correlation with the occurrence of patellar femoral arthritis^19^. The cross‐sectional shape and articular surface of patella flatten after patella instability in growing rabbits, indicating that patella instability may lead to patella dysplasia^20^. Conversely, since patella development is affected in developing patients with femoral trochlear dysplasia, improved alignment may reduce or ameliorate this effect, which is an additional basis for considering soft tissue surgery for patella dislocation in developing children. However, the current research on this is still shallow, and there are not a large number of high‐quality related reports, in particular those detailing soft tissue surgery results. On the other hand, the course of bony procedures can damage the proximal tibia and the distal femur of the child, which is very dangerous and can lead to premature closure of the epiphysis, which in undesirable. Therefore, the hypothesis is that patella development can be affected by restoring the normal biomechanics of the patellofemoral joint.

The aim of this prospective study was: (i) to investigate the changes in patella morphology following soft tissue surgical correction for recurrent patellar dislocation in children; (ii) to observe and compare the effects of surgical interventions; and (iii) to provide a reference for children with recurrent patellofemoral dislocation to restore their normal anatomical structure of patellofemoral joint in a timely and effective manner.

## Patients and Methods

The study had ethical approval and all patients gave informed consent. The clinical trial registration number was ChiCTR2100051906.

**Fig 1:**
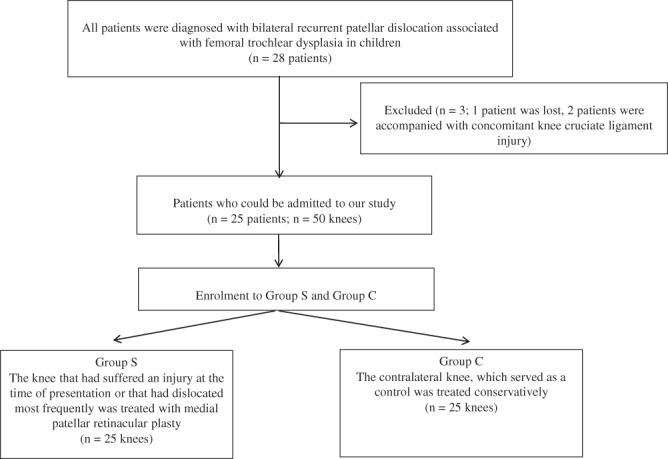
Consolidated Standards of Reporting Trials (CONSORT) flowchart of patient selection.

The Consolidated Standards of Reporting Trials (CONSORT) flowchart showing the selection of patients was shown in Fig. 1. A total of 25 patients, with a mean age of 8.4 years (range 7 to 10 years) were enrolled. All patients had bilateral recurrent patellar dislocation associated with trochlear dysplasia. The knee that had suffered an injury at the time of presentation or that had dislocated most frequently was treated with medial patellar retinacular plasty (surgery group). The contralateral knee, which served as a control, was treated conservatively (conservative group). All patients were treated either surgically or conservatively between November 2007 and December 2012, with a mean follow‐up of 60.8 months (range 48 to 75 months). All patients had CT scans preoperatively and at the final follow‐up, to assess the stability of the patellofemoral joint on axial slices and the patellar morphology on a particular axial image which was established at the point with the greatest patellar width based on measurements on axial slices^20^. In addition, the function of the knee joint was evaluated using the apprehension test^21^ and subjective evaluation^22^.

The inclusion criteria were: (i) bilateral recurrent patellar dislocation (recurrent being defined as more than one traumatic episode involving disruption of the normal position of the patella within the femoral groove^23^); (ii) open growth plates (as no changes in bone morphology occur after physeal closure^19^); (iii) Dejour A (shallow trochlea >150°) as described by Dejour *et al*.^21^, ^22^, ^24^, ^25^; and (iv) medial patellofemoral ligament (MPFL) injury.

The exclusion criteria were: (i) closed physes; (ii) a sulcus angle (SA) of <150° (an angle of >150° suggests trochlear dysplasia in children^7^, ^26^); (iii) a high‐grade trochlear dysplasia (Dejour B, C, and D)^21^, ^22^, ^24^, ^25^ (when patellar dislocation is accompanied by severe trochlear dysplasia, simple soft‐tissue balancing procedures cannot achieve satisfactory results, and artificial creation of a femoral sulcus is often required^27^); (iv) concomitant cruciate ligament or collateral ligament injury; and (v) rheumatoid arthritis or osteonecrosis with cartilage damage of greater than grade II^28^.

### Operative Technique

Arthroscopic exploration was performed to assess and address any possible chondral lesions and concomitant pathology before performing medial patellar retinaculum plasty in all patients. A force‐directed medial shift of the patella of less than one‐fourth the width of the patella indicates overtension of the lateral retinaculum structure, and in such cases, lateral retinacular release was performed^29^.

**Fig 2:**
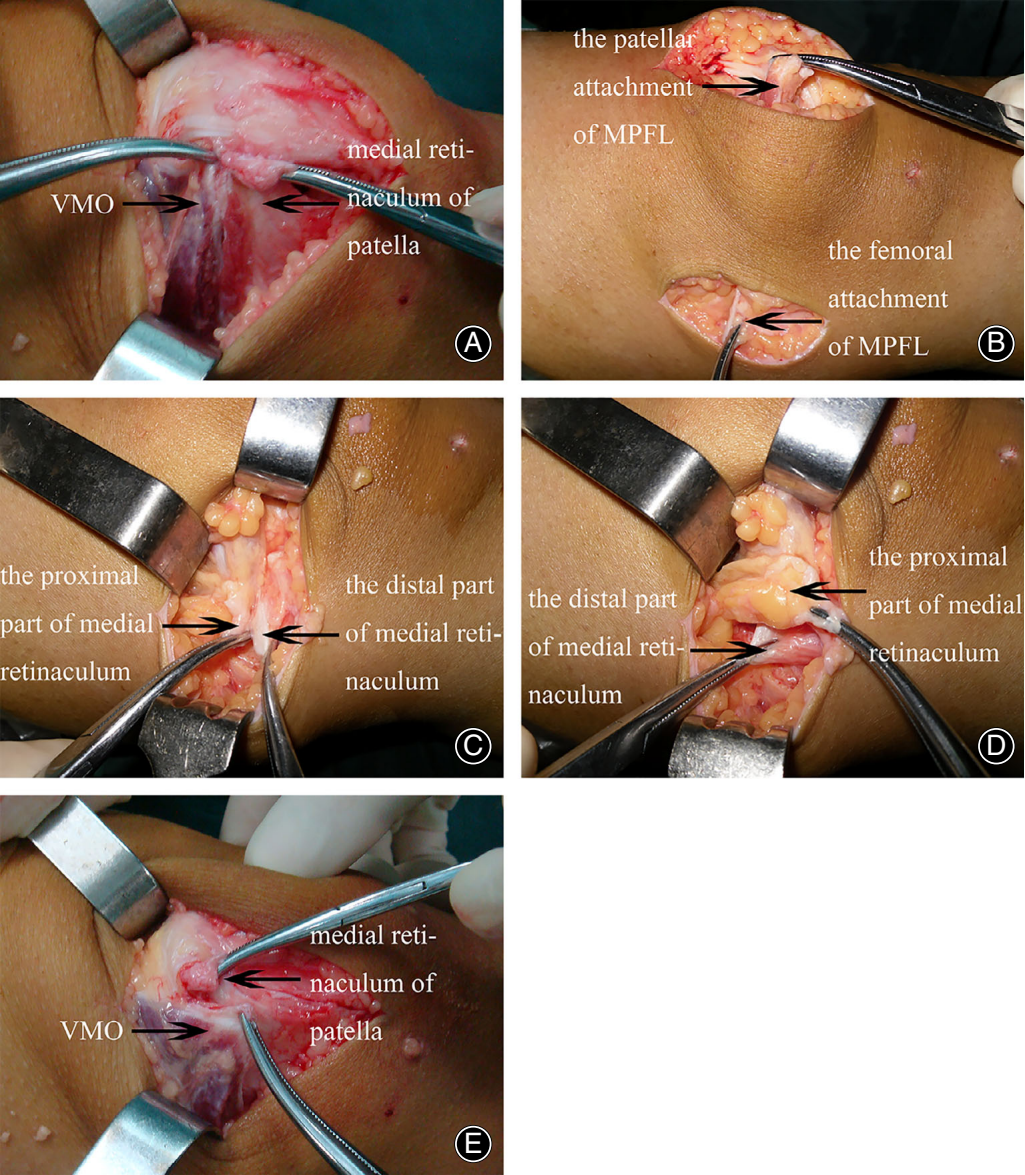
(A) A transverse incision was made at the junction of the vastus medialis obliquus (VMO) and the medial retinaculum. (B) The femoral attachment of medial patellofemoral ligament (MPFL) was dissected. (C) A transverse dissection was made to divide the medial retinaculum into two parts, a distal part, including both medial retinaculum and MPFL, and a proximal part, only including medial retinaculum. (D) The distal part of medial retinaculum was advanced proximally behind the medial femoral condyle and the proximal part of retinaculum, and then advanced distally over the retensioned distal part of medial retinaculum. (E) The isolated medial retinaculum of the patella was advanced proximally and laterally near to the upper pole of the patella, and then vastus medialis was advanced distally and laterally to the patella.

**Fig 3:**
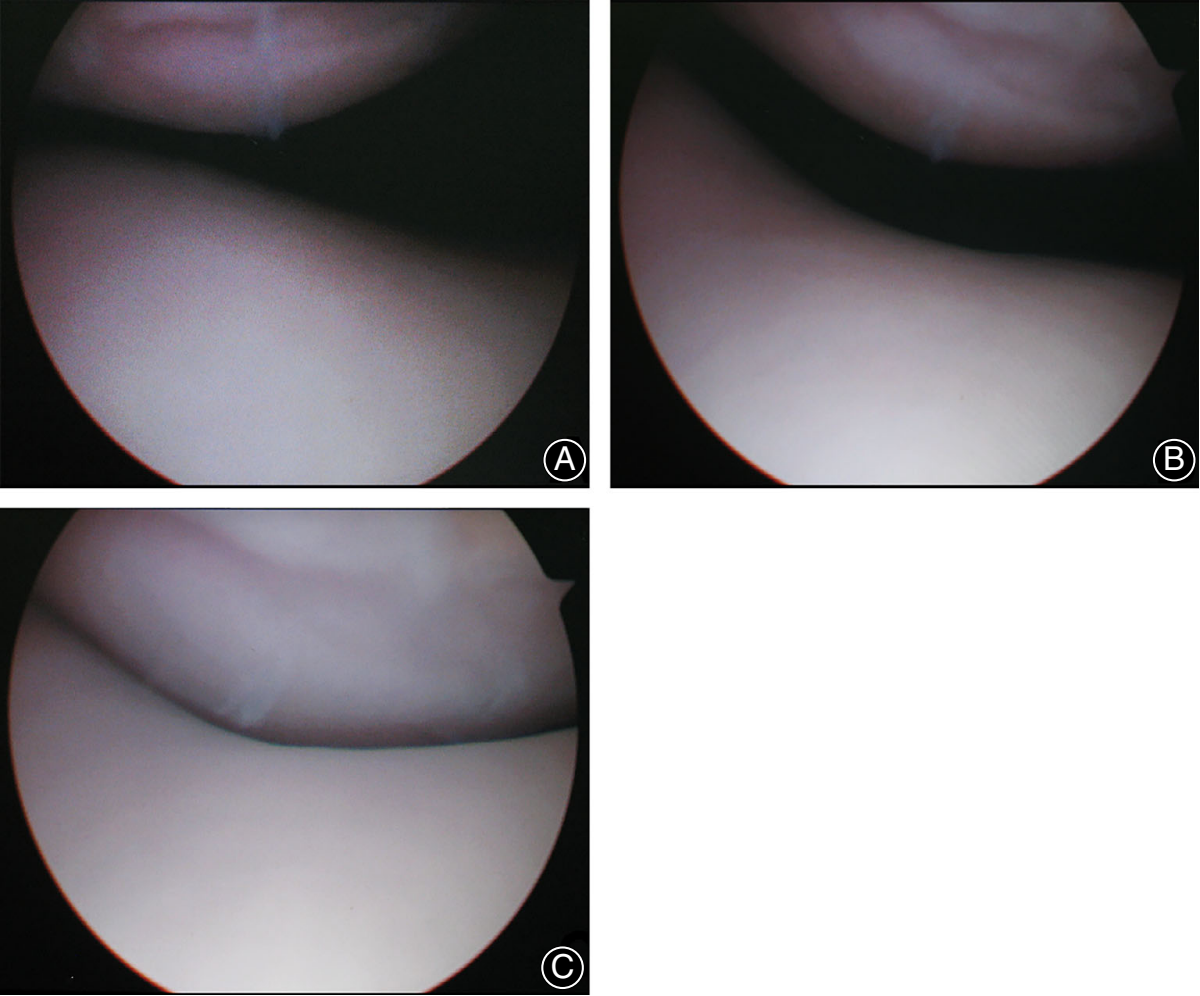
(A) Preoperative appearance of patellar dislocation. (B) Postoperative fine congruence of patellofemoral joint with the knee in full extension. (C) The patella stabilized to the trochlea at 10° flexion.

In surgery group, arthroscopic lateral retinacular release was performed in six patients. Under general anesthesia, the patients were in the supine position, and the operation was performed through a lateral incision. After removing the arthroscope, the concrete surgical procedure was as follows: a transverse incision was made at the junction of the vastus medialis obliquus (VMO) and the medial retinaculum. The femoral attachment of MPFL was dissected. A transverse dissection was made to divide the medial retinaculum into two parts: a distal part, including both medial retinaculum and MPFL, and a proximal part, only including medial retinaculum. The distal part of medial retinaculum was advanced proximally behind the medial femoral condyle and the proximal part of retinaculum, and then advanced distally over the retensioned distal part of medial retinaculum (Fig. 2). The isolated medial retinaculum of the patella was advanced proximally and laterally near to the upper pole of the patella, and then vastus medialis was advanced distally and laterally to the patella. Before the final tensioning, patellar tracking was observed manually, the patellofemoral congruence in full extension was noted and the tracking was checked throughout flexion arthroscopically and the tension of the medial retinaculum and vastus medialis (VM) was adjusted appropriately and the two sections were sutured together on the medial side of the patella (Fig. 3). Finally, the overlapping tissues (including the medial retinaculum, VM, and proximal and distal sections of the medial retinaculum) were sutured together with PDS‐1 sutures.

### Conservative Management

Implementation of the conservative treatment programme began at the same time as the surgery on the other knee. Conservative management in conservative group included immobilization with a patellar brace (Tru‐Pull Advanced System Brace; DJO Global, Vista, California)^30^, ^31^, ^32^ and physical therapy beginning with isometric quadriceps exercises and progressing through closed and open chain rehabilitation^33^, ^34^, ^35^. The patellar brace was helpful in controlling lateral instability and maintaining a stretch in the tight lateral retinaculum, thus enhancing the rehabilitation programme^30^, ^31^, ^32^, and the physical therapy can improve quadriceps strength and range of motion^33^, ^34^, ^35^. Osternig and Robertson^36^ reported that prophylactic knee bracing can actually alter neuromuscular control around the joint. This suggested that true modification of patellar tracking may occur with brace use. In addition, McConnell^37^ reported good success in patients with patellar instability using specific muscle strengthening and taping techniques to modify patellar tracking for a 12‐month treatment period. Therefore, the study involved fixed treatment with the patellar brace for at least 12 months, and physical therapy was almost identical in these patients and was performed every day.

### Assessment

#### Patellar apprehension test

After the patients had straightened the knee, the surgeon pushed the patella outwards. If the patient had intense pain or fear, the patella fear test will be positive.

#### Tibial tuberosity‐trochlear groove (TT‐TG) distance

TT‐TG played a decisive role in the evaluation of patellar femoral joint disease. Tibial osteotomy and medialization had been recommended in the literature for TT‐TG distance over 20 mm.

#### Congruence angle

The angle represented the relative position of the patella and the femur. Usually, the lower pole of the patella was located inside the angle bisector, that was, the angle was normal negative.

#### Patellar tilt angle

An increase in this angle indicated an increase in the inclination of the patella.

#### Patellar lateral shift

The inner edge of the patella being close, on, or beyond the vertical line is normal; away from the vertical line indicated the patella had shifted.

#### Patellar width (PW)

The length between the most medial edge and the most lateral edge of the patella as the baseline.

#### Wiberg index (WI)

The lower the Wiberg index, the less stable the patella.

#### The ratio of length of lateral patella to medial patella (EPIP)

The length of the lateral and medial facet was measured, and the facet ratio was calculated.

#### Wiberg angle (WA)

The angle between the slopes of the medial and lateral patella.

#### Patellar apprehension test and CT

**Fig 4:**
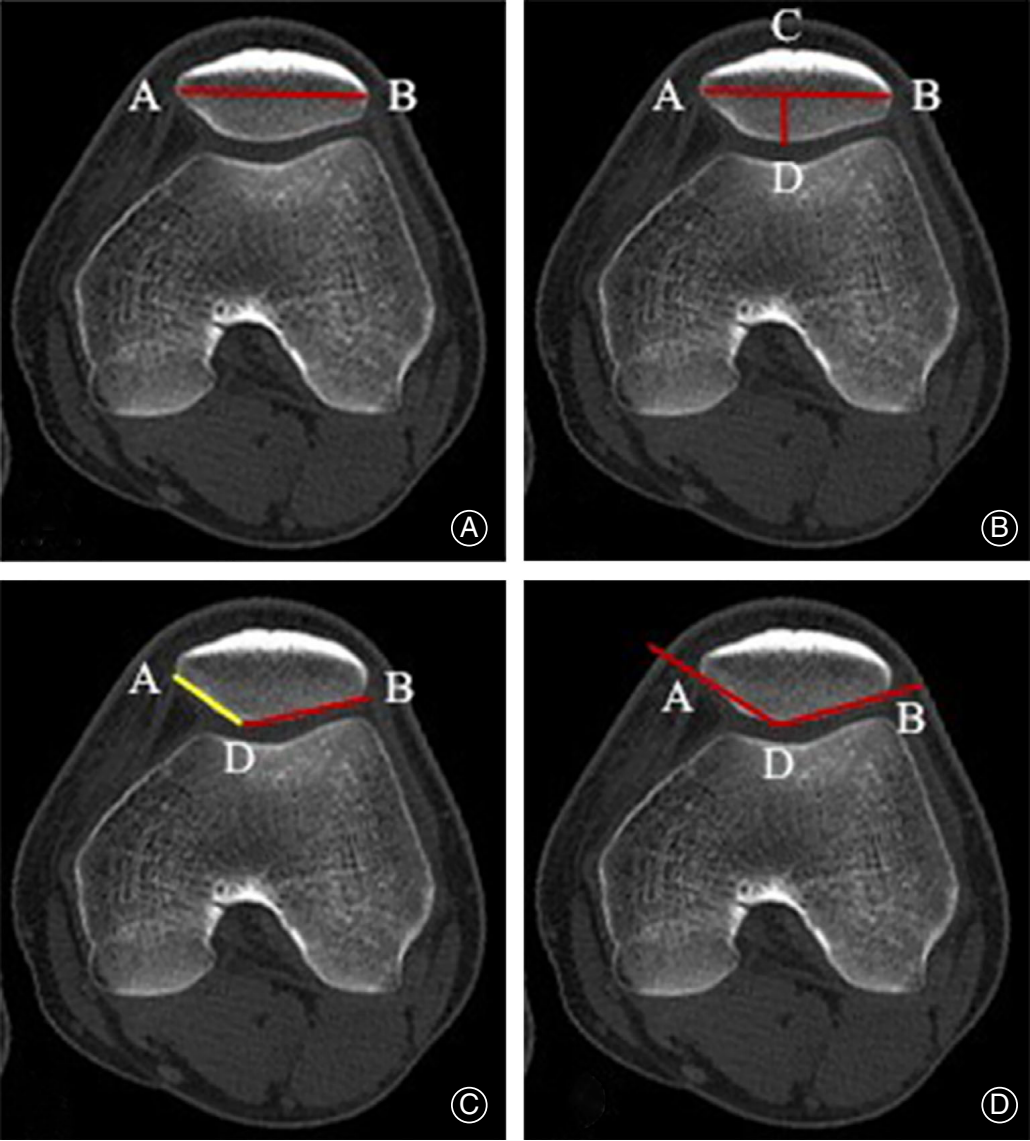
(A) Patellar width (AB). (B) Patellar thickness (CD) and Wiberg index (BC/AB). (C) The ratio of length of lateral patella to medial patella (BD/AD). (D) Wiberg angle (∠ADB).

**Fig 5:**
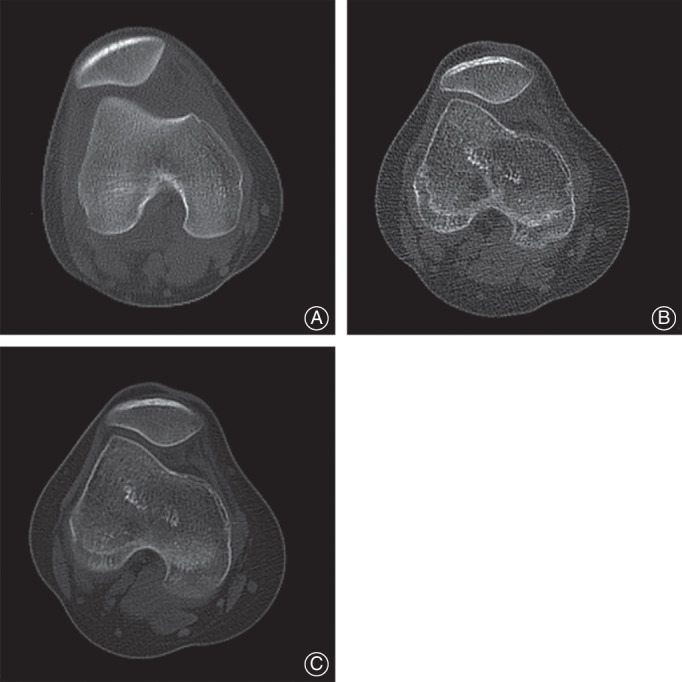
(A) Preoperative appearance of patellar dislocation. (B) Postoperative appearance of patellofemoral joint with the knee followed up 1 year later.

The diagnosis of patellar dislocation was confirmed by a patellar apprehension test and CT of the patellofemoral joint with the non‐weight‐bearing knee in full extension. In addition, preoperatively and at the last follow‐up, all patients underwent CT examination to assess the stability of the patellofemoral joint on axial slices and the patellar morphologic characteristics on a particular axial image which was established at the point with the greatest patellar width based on measurements on axial slices^20^, ^37^, ^38^. The methods used for the evaluation of patellar morphology were shown in Table 1 and Fig. 4, ^20^, ^37^, ^38^. Preoperative appearance of patellar dislocation and postoperative appearance of patellofemoral joint with the knee were shown in Fig. 5. All data were measured using Sante DICOM Viewer Free(64‐bit) verson 5.2 (Santesoft, Inc. Athens, Greece), which had an accuracy of 0.01° for angles and 0.01 mm for distance^39^. In order to minimize errors of measurement, all measurements were performed under the same conditions by two authors (KF and YZ). After an interval of 2 weeks, one measured the 25 samples again and the intra‐ and inter‐observer reliabilities were determined using intra‐observer and inter‐observer intra‐class correlation coefficients (ICCs).

**TABLE 1:** Description of measurements according to Fig. 4

Patellar morphological characteristics	
Patellar width (PW)^13^, ^24^, ^25^	The length between the most medial edge (A) and the most lateral edge (B) of the patella as the baseline (AB).
Patellar thickness (PT)^13^, ^24^, ^25^	The posterior patellar edge farthest from the baseline was defined as point D. The thickness of the patella was measured by the length of line CD vertical to the baseline.
Wiberg index (WI)^13^, ^25^	The Wiberg index (length of BC/length of AB) was calculated.
The ratio of length of lateral patella to medial patella (EPIP)^25^	The length of the lateral (BD) and medial (AD) facet were measured and the facet ratio ([BD] / [AD]) was calculated.
Wiberg angle (WA)^13^, ^25^	The angle (∠ADB) between the slopes of the medial and lateral patella.

The apprehension test was also used^40^ and the statement was recorded which best described the results of the treatment as described by Drez *et al*.^41^: (i) the knee had markedly improved, and returned to all activities; (ii) the knee had improved, but there was still occasional discomfort or problems in sports activities; (iii) the knee had improved, but the patient was still unable to return to sports activities; and (iv) the knee was not better or was worse than before surgery. The response yielded a subjective evaluation of excellent, good, fair, or poor, respectively. In addition, overall the patella form was rated according to the classification of Wiberg^42^ on a particular axial image which was established at the point with the greatest patellar width^20^, ^37^, ^38^. And the trochlea form was rated according to the classification of Dejour^21^, ^22^, ^24^ on a particular axial image which was established at the point with the greatest epicondylar width^19^, ^43^.

### Statistical Analysis

Statistical analysis was performed using the Statistical Package for the Social Sciences (SPSS) version 16.0 (SPSS, Chicago, Illinois). The Kolmogorov–Smirnov test was used to test the normality of numerical data. Levene's test was used to assess the homogeneity of the data. All numerical variables showed a normal distribution or equal variance. Differences between the two groups (evaluation indexes of stability of the patellofemoral joint and patella morphology) were analyzed with a two‐sample Student's *t*‐test. The apprehension sign, subjective scores, patellar types and trochlear types were analyzed using Pearson's chi‐squared test. Numerical data were shown as mean and standard deviation, and categorical data as numbers with percentages. The test level α = 0.05.

## Results

The prospective cohort study was performed between November 2007 and December 2012. Finally, 25 cases, with the mean age of 8.4 years (range 7 to 10 years), were admitted to the study. All patients were diagnosed as bilateral recurrent patellar dislocation associated with femoral trochlear dysplasia. The knee that had suffered an injury or dislocated most frequently was treated with medial patellar retinacular plasty (surgery group). The contralateral knee, which served as a control was treated conservatively (conservative group). The mean follow‐up was 60.8 months (range 48 to 75 months).

The intra‐class and inter‐class correlation coefficients were high for all measurements (Table 2).

**TABLE 2:** Intra‐observer and inter‐observer agreement of geometric measurements with 95% confidence intervals

Measurement	Intra‐observer			Inter‐observer		
	ICC	95%*CI* for ICC		*ICC*	95%*CI* for ICC	
		Lower	Upper		Lower	Upper
Pre‐surgery group‐TT‐TG	0.941	0.866	0.974	0.837	0.654	0.928
Pre‐surgery group‐CA	0.946	0.876	0.977	0.847	0.673	0.932
Pre‐surgery group‐PTA	0.949	0.883	0.978	0.808	0.548	0.919
Pre‐surgery group‐PLS	0.956	0.896	0.981	0.915	0.811	0.963
Pre‐surgery group‐PW	0.916	0.812	0.963	0.956	0.896	0.981
Pre‐surgery group‐PT	0.872	0.697	0.946	0.772	0535	0.897
Pre‐surgery group‐WI	0.938	0.853	0.974	0890	0.758	0.952
Pre‐surgery group‐EPIP	0.872	0.697	0.946	0.883	0.774	0.949
Pre‐surgery group‐WA	0.773	0.535	0.897	0.938	0.953	0.974
Post‐surgery group‐TT‐TG	0.954	0.895	0.980	0.917	0.805	0.965
Post‐surgery group‐CA	0.735	0.375	0.888	0.847	0.674	0.932
Post‐surgery group‐PTA	0.937	0.850	0.973	0.923	0.828	0.967
Post‐surgery group‐PLS	0.982	0.957	0.992	0.960	0.906	0.983
Post‐surgery group‐PW	0.881	0.740	0.948	0.964	0.918	0.985
Post‐surgery group‐PT	0.963	0.914	0.984	0.932	0.846	0.970
Post‐surgery group‐WI	0.932	0.846	0.970	0.927	0.837	0.969
Post‐surgery group‐EPIP	0.929	0.841	0.969	0.962	0.911	0.984
Post‐surgery group‐WA	0.958	0.900	0.982	0.919	0.819	0.965
Pre‐conservative group‐TT‐TG	0.977	0.945	0.990	0.849	0.677	0.933
Pre‐conservative group‐CA	0.834	0.648	0.926	0.918	0.807	0.965
Pre‐conservative group‐PTA	0.983	0.959	0.993	0.906	0.791	0.959
Pre‐conservative group‐PLS	0.966	0.922	0.985	0.950	0.883	0.979
Pre‐conservative group‐PW	0.967	0.923	0.986	0.900	0.779	0.956
Pre‐conservative group‐PT	0.983	0.960	0.993	0.947	0.876	0.978
Pre‐conservative group‐WI	0.902	0.783	0.957	0.948	0.878	0.978
Pre‐conservative group‐EPIP	0.904	0.788	0.958	0.901	0.782	0.957
Pre‐conservative group‐WA	0.948	0.877	0.978	0.950	0.881	0.979
Post‐conservative group‐TT‐TG	0.909	0.787	0.962	0.846	0.672	0.932
Post‐conservative group‐CA	0.911	0.791	0.962	0.917	0.804	0.965
Post‐conservative group‐PTA	0.929	0.833	0.970	0.949	0.880	0.978
Post‐conservative group‐PLS	0.868	0.717	0.942	0.903	0.786	0.958
Post‐conservative group‐PW	0.868	0.714	0.942	0.892	0.763	0.953
Post‐conservative group‐PT	0.929	0.833	0.970	0.943	0.865	0.976
Post‐conservative group‐WI	0.944	0.869	0.976	0.926	0.826	0.969
Post‐conservative group‐EPIP	0.895	0.768	0.954	0.875	0.727	0.945
Post‐conservative group‐WA	0.933	0.842	0.972	0.863	0.704	0.939

EPIP, the ratio of length of lateral patella to medial patella; ICC, intra‐class correlation coefficients; Post, postoperative; Pre, preoperative; PT, patellar thickness; PW, patellar width; WA, Wiberg angle; WI, Wiberg index.

### Evaluation Indexes of Patellofemoral Joint Stability

Preoperatively, there were also no statistically significant differences between the patellofemoral joint stability in the two groups (tibial tuberosity‐trochlear groove distance, *P* > 0.05; congruence angle, *P* > 0.05; patellar tilt angle, *P* > 0.05; patellar lateral shift, *P* > 0.05) (Table 3). At the final follow‐up, these parameters were significantly different between the two groups, such as the tibial tuberosity‐trochlear groove distance (surgery group, 10.35 mm [SD 1.65]; conservative group, 15.82 mm [SD 1.73]; *P* < 0.05), the congruence angle (surgery group, 9.89° [SD 1.47°]; conservative group, 21.97° [SD 3.51°]; *P* < 0.05), the patellar tilt angle (surgery group, 8.16° [SD 1.52°]; conservative group, 14.73° [SD 2.68°]; *P* < 0.05) and the patellar lateral shift (surgery group, 9.59 mm [SD 2.71]; conservative group, 17.85 mm [SD 3.84]; *P* < 0.05) (Table 4). Additionally, 15 patients in conservative group had recurrent patellar dislocation. However, only two patients in surgery group had a patellar lateral shift that exceeded 1.5 cm with a hard end point for the apprehension test, with a significant difference between the two groups. The subjective questionnaire revealed 14 (56%) excellent results in surgery group and zero (0%) in conservative group; zero (0%) patients in surgery group and 10 (40%) patients in conservative group answered “poor,” which was a significant difference between the two groups.

**TABLE 3:** Preoperative evaluation of knee function

Indexes	Surgery group	Conservative group	*t* values	*P* values
CT				
TT‐TG (mm)	15.32 ± 1.56	15.16 ± 1.44	0.36	>0.05
CA (°)	21.75 ± 2.79	22.13 ± 2.76	−0.47	>0.05
PTA (°)	14.69 ± 2.53	14.32 ± 2.61	0.50	>0.05
PLS (mm)	18.72 ± 3.05	19.13 ± 2.96	−0.47	>0.05

CA, congruence angle; PLS, patellar lateral shift; PTA, patellar tilt angle; TT‐TG, tibial tuberosity‐trochlear groove distance.

**TABLE 4:** Follow‐up results of knee function

Indexes	Surgery group	Conservative group	*t* values	*P* values
CT				
TT‐TG (mm)	10.35 ± 1.65	15.82 ± 1.73	−11.21	<0.05
CA (°)	9.89 ± 1.47	21.97 ± 3.51	−15.57	<0.05
PTA (°)	8.16 ± 1.52	14.73 ± 2.68	−10.44	<0.05
PLS (mm)	9.59 ± 2.71	17.85 ± 3.84	−8.61	<0.05
Apprehension sign [n/N (%)]				
<1.5 cm	23/25 (92%)	4/25 (16%)		
>1.5 cm	2/25 (8%)	21/25 (84%)		
Subjective questionnaire [n/N (%)]				
Excellent	14/25 (56%)	0/25 (0)		
Good	9/25 (36%)	3/25 (12%)		
Fair	2/25 (8%)	12/25 (48%)		
Poor	0/25 (0)	10/25 (40%)		

CA, congruence angle; PLS, patellar lateral shift; PTA, patellar tilt angle; TT‐TG, tibial tuberosity‐trochlear groove distance.

### Evaluation Indexes of Patellar Morphology

Preoperatively, the data regarding the patellar morphological characteristics were not significantly different between the groups (*P* > 0.05) (Table 5). Many measurements showed significant differences between the two groups at the last follow‐up (Table 6). Significant differences were seen in well‐known measurements such as the patellar width (PW) (surgery group, 40.58 mm [SD 1.26]; conservative group, 36.41 mm [SD 1.17]; *P* < 0.05), patellar thickness (PT) (surgery group, 11.59 mm [SD 0.74]; conservative group, 9.38 mm [SD 0.56]; *P* < 0.05), and Wiberg index (WI) (surgery group, 0.54 [SD 0.06]; conservative group, 0.72 [SD 0.08]; *P* < 0.05). However, lesser‐known measurements such as the ratio of length of lateral patella to medial patella (EPIP) (surgery group, 1.26 [SD 0.17]; conservative group, 1.69 [SD 0.21]; *P* < 0.05), which was a measurement of facet asymmetry, were also significantly different between the groups. However, the Wiberg angle (WA) was not significantly different between the two groups (surgery group, 128.63° [SD 9.05°]; conservative group, 125.47° [SD 13.96°]; *P* > 0.05).

**TABLE 5:** Preoperative evaluation of patellar morphologic characteristics

Indexes	Surgery group	Conservative group	*t* values	*P* values
CT				
PW (mm)	31.78 ± 1.49	32.56 ± 1.57	−1.78	>0.05
PT (mm)	7.51 ± 1.26	7.64 ± 1.32	−0.34	>0.05
WI	0.62 ± 0.08	0.61 ± 0.07	0.37	>0.05
EPIP	1.37 ± 0.25	1.34 ± 0.23	0.46	>0.05
WA (°)	134.72 ± 7.09	135.45 ± 5.86	−0.39	>0.05

EPIP, the ratio of length of lateral patella to medial patella; PT, patellar thickness; PW, patellar width; WA, Wiberg angle; WI, Wiberg index.

**TABLE 6:** Follow‐up results of patellar morphologic characteristics

Indexes	Surgery group	Conservative group	*t* value	*P* value
CT				
PW (mm)	40.58 ± 1.26	36.41 ± 1.17	11.87	<0.05
PT (mm)	11.59 ± 0.74	9.38 ± 0.56	11.72	<0.05
WI	0.54 ± 0.06	0.72 ± 0.08	−9.34	<0.05
EPIP	1.26 ± 0.17	1.69 ± 0.21	−7.76	<0.05
WA (°)	128.63 ± 9.05	125.47 ± 13.96	0.93	>0.05

EPIP, the ratio of length of lateral patella to medial patella; PW, patellar width; PT, patellar thickness; WA, Wiberg angle; WI, Wiberg index.

### The Classification of Patella and Trochlea Form

Preoperatively, there were also no statistically significant differences between the classification of patella and trochlea form in the two groups (*P* > 0.05). (Tables 7 and 8). At the final follow‐up, the patella form in surgery group was classified as Wiberg‐I in 10 (40%) cases, Wiberg‐II in 13 (52%) cases, and Wiberg‐III in two (8%) cases; in conservative group was classified as Wiberg‐I in zero (0%) cases, Wiberg‐II in 14 (56%) cases, and Wiberg‐III in 11 (44%) cases, which was a significant difference between the two groups. And the trochlea form in surgery group was classified as normal in 10 (40%) cases, Dejour‐A in 13 (52%) cases, Dejour‐B in two (8%) cases, and Dejour‐C/D in zero (0%) cases; in conservative group, the trochlea form was classified as normal in zero (0%) cases, Dejour‐A in four (16%) cases, Dejour‐B in nine (36%) cases, Dejour‐C in eight (32%) cases, and Dejour‐D in four (16%) cases, which was a significant difference between the two groups.

**TABLE 7:** Patellar types[n/N (%)]

Type	Wiberg classification		
	I	II	III
Pre‐surgery group	4/25 (16%)	21/25 (84%)	0/25 (36%)
Pre‐conservative group	5/25 (20%)	20/25 (80%)	0/25 (36%)
Post‐surgery group	10/25 (40%)	13/25 (52%)	2/25 (8%)
Post‐conservative group	0/25 (0)	14/25 (56%)	11/25 (44%)

Pre, preoperative; Post, postoperative.

**TABLE 8:** Trochlear types[n/N (%)]

Type	Dejour classification				
	N	A	B	C	D
Pre‐surgery group	0/25 (0)	9/25 (36%)	16/25 (64%)	0/25 (0)	0/25 (0)
Pre‐conservative group	0/25 (0)	11/25 (44%)	14/25 (56%)	0/25 (0)	0/25 (0)
Post‐surgery group	10/25 (40%)	13/25 (52%)	2/25 (8%)	0/25 (0)	0/25 (0)
Post‐conservative group	0/25 (0)	4/25 (16%)	9/25 (36%)	8/25 (32%)	4/25 (16%)

Pre, preoperative; Post, postoperative.

No patellar redislocation case was found in the follow‐up.

## Discussion

Compared with conservative management, it had been found that the stability of the patellofemoral joint can be significantly improved by early (before epiphyseal closure) soft tissue operative intervention, with decreased recurrent instability. In the growing child, soft tissue methods were the only logical treatment options because bony procedures injure the proximal tibial physis and distal femoral physis, potentially leading to premature closure^18^, ^44^. Lesions of the MPFL were found in almost all patients with patellar dislocation^45^. Balcarek *et al*.^46^ reported that MPFL injury occurred in 98.6% of patients with patellar dislocation. Correlation studies had confirmed that the MPFL contributes about 53% to 67% of the total medial restraining force as a distinct restraining structure in the second layer of the medial soft tissues^47^, ^48^. Therefore, all patients in surgery group underwent surgical treatment (medial patellar retinaculum plasty). Ma *et al*.^17^, ^40^, ^49^ mainly concentrated on repair of the MPFL and proved that this procedure can restore the anatomical function of the MPFL almost to its full capacity as well as the static and dynamic stability of the patella. However, conservative treatment cannot fundamentally solve the patellar imbalance induced by a loose medial patellar retinaculum and tight lateral patellar retinaculum. Therefore, while many patients treated nonoperatively did not develop recurrent patellar dislocation^50^, long‐term studies demonstrated recurrent dislocation in 30% to 50% of patients, with others reporting subjective instability, pain, and persistent disability^51^, ^52^.

Patellar morphological characteristics were a major aspect of the study and yielded interesting results. The mean PWs were significantly different at the final follow‐up (surgery group, from 31.78 mm to 40.58 mm; conservative group, from 32.56 mm to 36.41 mm). Fucentese *et al*.^38^ reported mean values of 42.00 mm and 38.27 mm in the transverse plane with the widest diameter of the patella for controls and patients with patellofemoral joint dislocation (PFJD), which was a significant difference between the two groups (*P* = 0.005). Therefore, the values were consistent with the results of Fucentese *et al*.^38^ at the final follow‐up.

The PT was also often used in the patellofemoral literature^20^, ^37^, ^38^. Fucentese *et al*.^38^ reported that a mean of >10.60 mm was normal and that <9.59 mm indicated pathological for patellofemoral instability, which was a significant difference between the two groups (*P* = 0.015). Values were between 7.51 mm and 11.59 mm in surgery group, between 7.64 mm and 9.38 mm in conservative group, which reflected the results of Fucentese *et al*.^38^ at the final follow‐up.

The WI was another classic measurement of patellar morphological characteristics^20^, ^38^. A mean of >0.60 for patients with patellofemoral instability and a mean of <0.54 for the controls were reported by Fucentese *et al*.^38^, which was a significant difference between the two groups (*P* = 0.013). The values (surgery group, from 0.62 to 0.54; conservative group, from 0.61 to 0.72) reflected the same trend of Fucentese *et al*.^38^ at the final follow‐up.

Facet asymmetry was an aspect of patellar dysplasia that has attracted little attention until recently^38^. The research showed that the length of the medial facet was shortened and the length of the lateral facet was essentially the same in comparison with surgery group, leading to an increased facet ratio in conservative group (surgery group, from 1.37 to 1.26; conservative group, from 1.34 to 1.69). Fucentese *et al*.^38^ reported that a mean of <1.15 is normal and that >1.42 indicates patellar dysplasia. Therefore, compared with conservative group, the mean EPIP in surgery group was closer to normal at the end of the study.

The WA has also received little attention and its clinical significance remains unclear. Jinghui *et al*.^20^ reported mean values of 131.1° and 148.8° for controls and experimental group with patellofemoral joint dislocation (PFJD) in the animal experiment of growing rabbits, which was a significant difference between the two groups (*P* < 0.05). However, Fucentese *et al*.^38^ measured the WA conservative group, 129.36° (SD 7.02°); surgery group, 125.64° (SD 11.80°); *P* = 0.254); no significant difference was found between controls and patients with patellar instability. Values for the WA of between 134.72° and 128.63° in surgery group and between 135.45° and 125.47° in conservative group reflected no significant difference at the final follow‐up, which was consistent with the results of Fucentese *et al*.^38^. Surprisingly, a flattening of the retropatellar Wiberg angle, which may be the consequence of a missing trochlear groove, was not found. This was unexpected since it has been shown that a high‐grade trochlear dysplasia (Dejour B, C, and D) corresponds with decreased sulcus angle^53^, ^54^. This can lead to severe patellar dysplasia (Wiberg III) corresponding with decreased Wiberg angle^42^. There was no statistical difference in the results, which may suggest that severe patellar dysplasia (Wiberg III) is present in the conservative treatment group.

Also, the patella type, classified according to Wiberg, showed a significant prevalence of type I and II patellae in surgery group and type II and III patellae in conservative group at the final follow‐up. Additionally, the trochlea type, classified according to Dejour, showed a significant prevalence of the normal and type A trochlea in surgery group and type B, C, and D trochlea in conservative group at the final follow‐up. It can be seen that the patella types were consistent with the measured data of patellar morphological characteristics.

Studies showed that patellofemoral dysplasia may be caused by patellofemoral dislocation, and the development of patella has attracted people's attention in recent years^55^. One important conclusion can be drawn from these results, that patellar morphology can be improved following early (before epiphyseal closure) soft tissue surgical correction of patellar instability associated with low‐grade trochlear dysplasia in children. In the patients of patellar instability, the key morphologic change of the patella was a decreased medial facet length^38^. This may explain the smaller patella, the increased Wiberg index, the basically consistent Wiberg angle, the increased facet ratio according with Servien *et al*.^56^, and the incidence of patella types II and III in the Wiberg classification^42^ in conservative group. But what exactly was the mechanism that induced the transformation of the decreased medial facet length remains unclear and will need to be further explored by future researchers. Perhaps this is because the appropriate stimulation of stress guides the development of the bone^7^, ^8^, ^19^. The external displacement of patella leaded to the disappearance of the normal matching relationship of the patellofemoral joint, resulting in abnormal force on the medial articular surface of the patella, which decreased or even did not bear, thus causing dysplasia of the medial articular surface of the patella (a decreased medial facet length)^55^. Therefore, for children with recurrent patellar dislocation, it was very necessary to restore the normal biomechanics of patellofemoral joint by soft tissue surgical correction in time.

This study had limitations. Firstly, CT can be used to describe the osseous contour which cannot be matched with the corresponding surface, which was covered with cartilage when viewed by MRI^37^, ^38^, ^43^. Secondly, single axial cuts on a CT scan cannot be used to elucidate the trochlear morphology. Additional axial slices were required to assess the geometry more accurately^43^. Moreover, the number of patients was low (25). More patients who meet the criteria are expected to be included in future analyses.

In conclusion, compared with conservative management, early surgical treatment using medial patellar retinacular plasty was the preferred method for decreasing recurrent instability and improving the outcomes in children with recurrent patellar instability associated with low‐grade trochlear dysplasia (Dejour A). The main finding was that the patellar morphology can be significantly improved by early (before epiphyseal closure) soft tissue surgical correction in children with patellar instability associated with low‐grade femoral trochlear dysplasia (Dejour A).

## Declarations

### Ethics Approval and Consent to Participate

The present study was approved by the Academic Ethics Committee of the Third Hospital of Hebei Medical University. The trial was conducted in accordance with the Declaration of Helsinki. All participants were provided information about the study and enrolled into the trial if they met all eligibility criteria and voluntarily signed an informed consent statement.

## Consent for Publication

Not applicable.

## Availability of Data and Materials

The datasets used during the current study are available from the corresponding author on reasonable request.

## Authors' Contributions

Fei Wang contributed to the conception of the study; Conglei Dong and Yanyang Wang contributed significantly to the analysis and wrote the manuscript; Wei Lin and Xiaobo Chen analyzed the numbers; Chenyue Xu and Maozheng Wei helped perform the analysis and create pictures with constructive discussions. The authors read and approved the final manuscript.
